# Immunohistochemical Evaluation of Integrin β6 Expression in Triple-Negative Breast Cancer as a Predictive Biomarker for Therapeutic and Diagnostic Radionuclides

**DOI:** 10.3390/biom16050706

**Published:** 2026-05-11

**Authors:** Muin Tuffaha, Wael Hananeh, Nikola Bangemann, Amro Tuffaha, Michael Starke

**Affiliations:** 1 Institute of Pathology, Medical University Lausitz-Carl-Thiem, 03048 Cottbus, Germany; 2 Department of Veterinary Pathology and Public Health, Faculty of Veterinary Medicine, Jordan University of Science and Technology, Irbid 22110, Jordan; 3 Department of Senology and Gynecological Oncology, Medical University Lausitz-Carl-Thiem, 03048 Cottbus, Germany; n.bangemann@mul-ct.de; 4 Medical Clinic, Pulmonology, Medical University Lausitz-Carl-Thiem, 03048 Cottbus, Germany; 5 Nuclear Medicine, Medical University Lausitz-Carl-Thiem, 03048 Cottbus, Germany

**Keywords:** integrin, breast cancer, triple negative, biomarker, radionuclides

## Abstract

Triple-negative breast cancer (TNBC) is an aggressive breast cancer subtype associated with limited therapeutic options and poor clinical outcomes. The aim of this research is to assess the prevalence, intensity, and distribution of integrin αvβ6 expression in TNBC using immunohistochemistry and to evaluate its potential as a predictive biomarker for αvβ6-targeted radionuclide therapy and other αvβ6-targeted theranostic approaches. Immunohistochemical analysis of integrin αvβ6 was performed on formalin-fixed, paraffin- embedded tumor samples from 48 patients with histologically confirmed TNBC. Staining intensity and the proportion of positive tumor cells were assessed using a semi-quantitative scoring system, and expression patterns were analyzed with regard to cellular localization and intratumoral heterogeneity. Moderate to strong integrin αvβ6 expression was observed in 43.8% of cases, with strong expression (≥50% of tumor cells) present in 25%. Expression was predominantly membranous, with occasional cytoplasmic staining, and demonstrated marked inter- and intratumoral heterogeneity. Integrin αvβ6 is frequently expressed in TNBC and represents a promising biomarker for patient selection in αvβ6-targeted radionuclide imaging and therapy. These findings provide a strong biological rationale for the clinical translation of integrin-targeted radioligands and support the development of personalized radiotheranostic strategies in TNBC.

## 1. Introduction

TNBC represents a clinically aggressive subtype of breast carcinoma defined by the absence of estrogen receptor, progesterone receptor, and human epidermal growth factor receptor 2 (HER2) expression [1]. Accounting for approximately 15–20% of all breast cancers, TNBC is characterized by a high propensity for early recurrence, rapid metastatic dissemination, and poor overall survival [2,3]. The lack of well-established molecular targets limits therapeutic options primarily to cytotoxic chemotherapy, with only a subset of patients benefiting from emerging immune checkpoint inhibitors [4,5]. These limitations underscore a critical unmet need for robust predictive biomarkers and novel molecularly targeted treatment strategies in TNBC. In this current study, TNBC was selected because of its limited therapeutic options and the previously reported association between integrin αvβ6 expression and aggressive disease biology in this subtype [6].

Integrin αvβ6 has emerged as a promising candidate in this context [7,8]. This heterodimeric, epithelium-restricted cell surface receptor consists of the integrin αvβ6 subunit paired with integrin αv [9]. Under physiological conditions, αvβ6 expression is minimal or absent in most adult epithelial tissues but is markedly upregulated in a variety of epithelial malignancies, including breast carcinoma [10]. In TNBC, increased expression of the β6 subunit has been associated with enhanced tumor cell migration, invasion, and survival, and correlates with aggressive clinical behavior and adverse prognosis [7,11]. Similar observations across multiple carcinoma types further support a central role for αvβ6 in tumor progression [12,13,14].

At the functional level, integrin αvβ6 contributes to tumorigenesis through several interconnected signaling pathways. It promotes extracellular matrix remodeling and tumor invasion via modulation of matrix metalloprotease activity and serves as a key activator of latent transforming growth factor-β (TGF-β) [8,15]. Subsequent activation of TGF-β signaling drives epithelial-to-mesenchymal transition (EMT), facilitates immune evasion, and contributes to therapeutic resistance. Recent preclinical studies have further linked αvβ6 expression in TNBC to the upregulation of TGF-β-responsive genes, including SOX4, as well as to immune suppressive pathways, highlighting its multifaceted role in tumor biology [16,17]. The tumor-restricted expression profile and functional relevance of αvβ6 make it an attractive target for molecular imaging and targeted therapy [18,19]. In recent years, several αvβ6-directed radiotracers have been developed for positron emission tomography (PET), including 68Ga-labeled compounds such as 68Ga-Trivehexin, which have demonstrated high specificity and favorable tumor uptake in αvβ6-expressing malignancies [20]. Early clinical data suggest that αvβ6-targeted PET/CT imaging is feasible in TNBC and may enable noninvasive assessment of target expression, thereby supporting its use as a diagnostic and theranostic biomarker [21].

Concurrently, advances in radiotheranostics have enabled the translation of such targeting strategies into therapeutic applications through the use of radionuclide-labeled ligands. In particular, β-emitting radionuclides such as lutetium-177 (^177^Lu) and α-emitting radionuclides such as actinium-225 (^225^Ac) and Astatine-211 (^211^At) can be conjugated to αvβ6-targeting peptides to deliver cytotoxic radiation selectively to tumor cells. Preclinical investigations of ^177^Lu-labeled αvβ6-targeted agents have demonstrated high binding affinity, favorable tumor retention, and significant antitumor efficacy, including prolonged survival in αvβ6-positive tumor models [20,22]. Despite these promising developments, a critical gap remains in translating these approaches into clinical practice, particularly with respect to defining the relationship between integrin αvβ6 expression in human TNBC tissues and the potential efficacy of integrin-targeted radioligand therapy.

In this study, we performed a systematic immunohistochemical analysis of integrin αvβ6 expression in formalin-fixed, paraffin-embedded tissue samples from primary TNBC. The aim was to determine the prevalence, intensity, and spatial distribution of integrin αvβ6 expression in tumor cells and to assess its suitability as a biomarker for integrin-targeted radiotheranostic approaches. In addition, we explored the radiobiological implications of αvβ6 expression patterns in the context of radionuclide therapy using both β-emitting (^177^Lu) and α-emitting (^225^Ac) or (^211^At) isotopes.

Comprehensive characterization of integrin αvβ6 expression at the tissue level may provide a critical link between molecular pathology and targeted radionuclide therapy. By establishing a biological rationale for patient stratification, this study seeks to support the development of integrin αvβ6-directed radioligand therapies and to advance precision oncology approaches in TNBC. To our knowledge, this represents the first systematic immunohistochemical evaluation of integrin αvβ6 expression in TNBC specifically aimed at informing the clinical applicability of ^177^Lu- and ^225^Ac- or ^211^At-labeled radiotherapeutics, with potential implications extending to other epithelial malignancies.

## 2. Materials and Methods

**Patient Cohort and Tissue Samples:** A total of 48 patients with histologically confirmed primary TNBC were included in this retrospective study. All cases were based exclusively on diagnostic core needle biopsy specimens obtained at initial presentation, prior to the initiation of any neoadjuvant or systemic therapy, thereby avoiding potential treatment-related alterations in biomarker expression. Clinicopathological data, including tumor characteristics and receptor status, were retrieved from institutional pathology records in accordance with standard diagnostic criteria.

**Tissue Processing and Immunohistochemistry:** Formalin-fixed, paraffin-embedded (FFPE) tissue blocks derived from core needle biopsies were used for immunohistochemical analysis. Serial sections of 1 µm thickness were cut and mounted on charged glass slides. After deparaffinization and rehydration, heat-induced antigen retrieval was performed using Tris-EDTA buffer (pH 9.0). Endogenous peroxidase activity was blocked prior to primary antibody incubation to minimize nonspecific staining.

Immunohistochemical detection of integrin β6 was performed using a rabbit monoclonal antibody (clone E4M9P; Cell Signaling Technology, Danvers, MA, USA) at a dilution of 1:200, recognizing endogenous human integrin αvβ6 protein. Immunoreactivity was visualized using a two-step horseradish peroxidase/diaminobenzidine (HRP/DAB) detection system, followed by hematoxylin counterstaining. Appropriate positive and negative controls were included in each staining run to ensure assay validity. To minimize inter-assay variability, all immunohistochemical staining procedures were performed under standardized conditions using the same reagents and an automated staining platform within a single experimental run.

**Evaluation of Integrin αvβ6 Expression:** Integrin αvβ6 expression was assessed in malignant epithelial cells with particular emphasis on membranous staining, consistent with the biological localization of the receptor. Cytoplasmic staining, when present, was noted but not considered the primary determinant of expression status. Staining intensity was evaluated semi-quantitatively and categorized as negative (0), weak (+), moderate (++), or strong (+++). In addition to staining intensity, the proportion of positively stained tumor cells was recorded to account for the extent and distribution of expression within the biopsy specimen. Strong expression was defined as membranous staining in ≥50% of tumor cells, whereas lower categories reflected decreasing intensity and/or extent of staining. Intratumoral heterogeneity was documented when variable staining patterns were observed within individual biopsy cores.

All slides were independently reviewed by experienced pathologists who were blinded to clinicopathological data to minimize observer bias. In cases of discrepant interpretation, a consensus evaluation was performed.

**Statistical Analysis:** Descriptive statistical methods were used to summarize the distribution of integrin αvβ6 expression levels across the cohort. Categorical variables were reported as absolute numbers and percentages. The analysis focused on the proportion of tumors exhibiting negative, weak, moderate, and strong expression, as well as the combined frequency of moderate-to-strong expression as a clinically relevant threshold for potential therapeutic targeting.

## 3. Results

A total of 48 diagnostic core biopsy specimens from patients with histologically confirmed primary TNBC were successfully evaluated for integrin αvβ6 expression by immunohistochemistry. Adequate tumor tissue for analysis was available in all cases, allowing for comprehensive assessment across the entire cohort.

**Figure 1:**
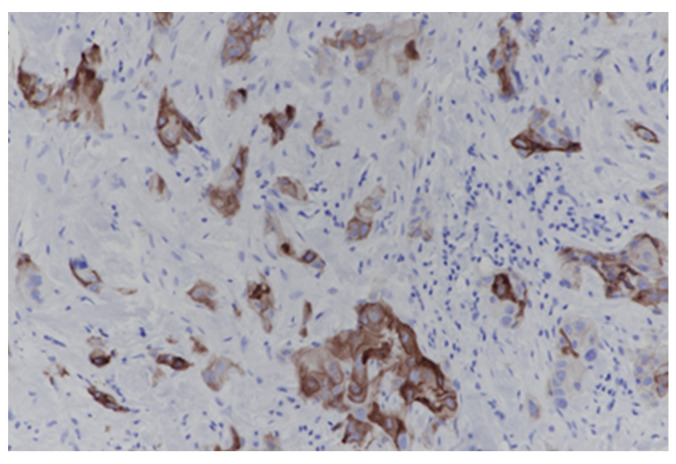
Breast core biopsy with intratumoral heterogeneity in integrin αvβ6 staining intensity. (immunostain, 200×).

Integrin αvβ6 immunoreactivity was predominantly localized to the cell membrane of malignant epithelial cells, consistent with the biological function of Integrin αvβ6 as a transmembrane adhesion receptor, and was occasionally accompanied by weaker cytoplasmic staining. The staining pattern was largely confined to neoplastic epithelial cells, with minimal or absent background in the surrounding stromal compartment, supporting the tumor-associated expression profile previously described for integrin. Considerable variability in staining intensity and the proportion of positive tumor cells was observed across specimens, and expression was therefore assessed using a semi-quantitative scoring system integrating both parameters. Tumors were stratified into four categories: strong (+++), moderate (++), weak (−), and negative (0) based on the extent and intensity of membranous staining. Strong expression was defined as intense membranous staining in ≥50% of tumor cells, whereas moderate expression corresponded to moderate to strong membranous staining in ≥50% of tumor cells. Weak expression was defined as membranous staining of any intensity in ≥10% of tumor cells, while negative expression was assigned to cases with complete absence of staining or staining present in <10% of tumor cells. Notably, variability in both staining intensity and distribution was observed not only between different tumors but also within individual tumor samples, reflecting presence of intratumoral heterogeneity in integrin αvβ6 expression (Figure 1). Such heterogeneity, characterized by intermixed regions of high and low membranous expression, may have important implications for both histopathologic interpretation and the performance of αvβ6-targeted molecular imaging or therapeutic strategies. A detailed overview of the distribution of expression levels is provided in Table 1.

**Figure 2:**
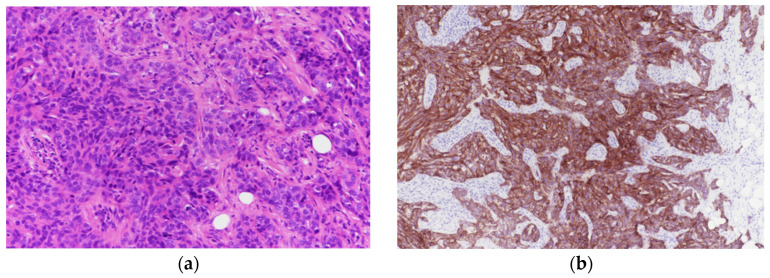
(**a**): Breast core biopsy infiltrated by triple-negative invasive ductal carcinoma (H&E 200×). (**b**): immunostaining of A. Tumor cells exhibiting strong membranous and cytoplasmic integrin expression in ~90% of tumor cells.

**Figure 3:**
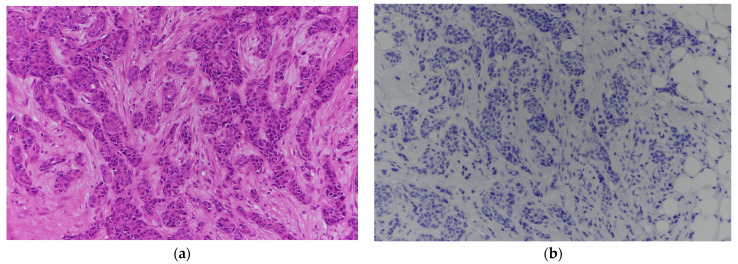
(**a**): Breast core biopsy infiltrated by triple-negative invasive ductal carcinoma (H&E 200×). (**b**): Immunostaining of A. Tumor cells exhibiting no membranous or cytoplasmic integrin expression in the entire section.

**Figure 4:**
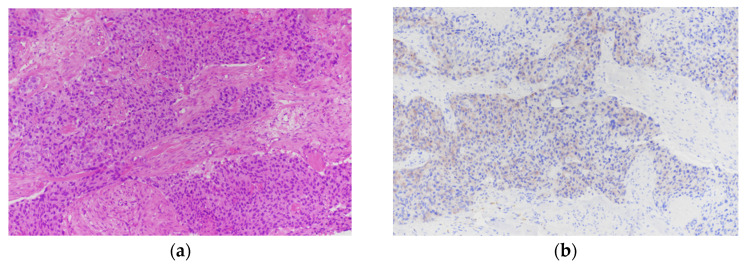
(**a**): Breast core biopsy infiltrated by triple-negative invasive ductal carcinoma (H&E 200×). (**b**): immunostaining of A. Tumor cells exhibiting weak integrin αvβ6 expression in ~25% of tumor cells.

**Figure 5:**
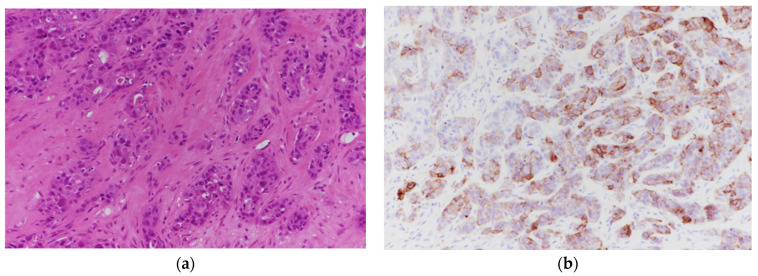
Aspiration pneumonia (**a**): Breast core biopsy infiltrated by triple-negative invasive ductal carcinoma (H&E 200×). (**b**): immunostaining of the A. Tumor cells exhibiting moderate to strong integrin αvβ6 expression in ~70% of tumor cells.

Strong integrin αvβ6 expression (≥50% of tumor cells with strong membranous staining) was observed in 12 of 48 cases (25.0%) (Figure 2a,b). In contrast, 16 of 48 tumors (33.3%) exhibited negative or minimal expression (Figure 3a,b). Weak expression was identified in 11 of 48 tumors (22.9%) (Figure 4a,b), while moderate expression was detected in 9 of 48 tumors (18.8%) (Figure 5a,b). Overall, moderate-to-strong integrin αvβ6 expression was present in 21 of 48 cases (43.8%), indicating that a substantial proportion of TNBCs exhibit biologically relevant levels of this target.

Descriptive analysis demonstrated a heterogeneous distribution of integrin αvβ6 expression across the cohort. Moderate-to-strong expression was observed in 43.8% of tumors, whereas 33.3% exhibited absent or minimal expression, highlighting substantial variability in target availability within TNBC. In 8 out of 12 strongly expressing cases (66.7%), areas of high expression were directly adjacent to negative or weak patches, with transition distances ranging from 50 to 300 µm, which is within the effective range of β-particles emitted by ^177^Lu (mean range ≈ 600 µm) but exceeds the range of α-particles (40–90 µm). Collectively, these findings indicate a heterogeneous but clinically relevant expression pattern of integrin αvβ6 in TNBC, with a distinct subset of tumors displaying high-level expression that may be amenable to integrin-targeted diagnostic and therapeutic approaches.

## 4. Discussion

Herein, we report the first systematic immunohistochemical analysis of integrin αvβ6 expression in a well-defined cohort of primary triple-negative breast cancer (TNBC) specimens specifically aimed at informing patient selection for αvβ6-targeted radionuclide therapy using β-emitting radionuclides such as Lutetium-177 (^177^Lu) and α-emitting radionuclides such as Actinium-225 (^225^Ac) and Astatine-211 (^211^At). Earlier studies have shown integrin αvβ6 overexpression in breast cancer and its association with aggressive behavior and poor prognosis; however, data focusing on TNBC is still limited. In a large immunohistochemical study of >2000 breast cancers, it was found that high expression of the integrin αvβ6was associated with a significantly reduced survival, with particularly poor outcomes in aggressive subtypes [12]. In accordance with these findings, we found moderate-to-strong membranous αvβ6 expression in 43.8% of TNBC cases. This study demonstrates that integrin αvβ6 is expressed in a substantial subset of newly diagnosed TNBC, with strong expression observed in 25% and moderate expression in 18.8% of cases. These findings support αvβ6 integrin’s role as a biologically and clinically relevant tumor-associated marker in this aggressive breast cancer subtype, which is characterized by limited therapeutic options and poor prognosis. The consistent detection of integrin αvβ6 by routine immunohistochemistry underscores the feasibility of incorporating this marker into standard pathological workflows and highlights its potential utility for patient stratification in the context of emerging targeted therapies. In this study, we build on previous prognostic studies and directly relate tissue-based expression data to the radiobiological requirements of β- and α-emitting radioligands to provide a translational basis for integrin-targeted radionuclide imaging and therapy in TNBC.

Integrin αvβ6 is also overexpressed in a wide range of epithelial cancers, and is frequently associated with aggressive tumor biology and poor clinical outcome. High expression has been consistently reported in pancreatic ductal adenocarcinoma [13], cholangiocarcinoma [23], colorectal cancer [24], gastric cancer [10], non-small-cell lung cancer [25], and head and neck squamous cell carcinomas [26]. In these tumors, αvβ6 mediates important oncogenic processes, including TGF-β activation, epithelial-to-mesenchymal transition, matrix remodeling, and metastatic dissemination [8]. Similar to the 43.8% prevalence of moderate to strong expression in our TNBC cohort, substantial intertumoral variability is often seen in other carcinomas, with strong membranous expression often restricted to subsets of tumors and absent or minimal in matched normal tissues. These results across various cancer types underscore the tumor-selective properties of αvβ6 and emphasize its utility as a pan-carcinoma biomarker, especially for radiotheranostic applications employing peptide-based radioligands. The analogous expression patterns indicate the potential to broaden αvβ6-targeted radionuclide therapies beyond TNBC to other challenging epithelial malignancies with significant unmet clinical need.

The observed heterogeneity expression pattern of integrin αvβ6 provides a compelling rationale for the development of αvβ6-targeted radiotheranostic approaches. Substantial intertumoral variability is often seen in other carcinomas [27,28]. Integrin-targeting strategies have gained increasing importance in molecular imaging and radionuclide therapy, and the presence of integrin αvβ6 expression in primary tumors-and potentially in metastatic lesions-supports its exploitation as a target for radioligand-based diagnostics and therapy [29,30,31]. In particular, radioligands labeled with β-emitting radionuclides such as ^177^Lu or high linear energy transfer (LET) α-emitting radionuclides such as ^225^Ac and ^211^At. offer distinct and potentially complementary therapeutic profiles [32,33]. In this setting, immunohistochemical assessment of integrin αvβ6 expression may serve as a practical and widely accessible biomarker to identify patients most likely to benefit from integrin-targeted radioligand therapy, complementing molecular imaging approaches and supporting personalized treatment selection.

**Figure 6:**
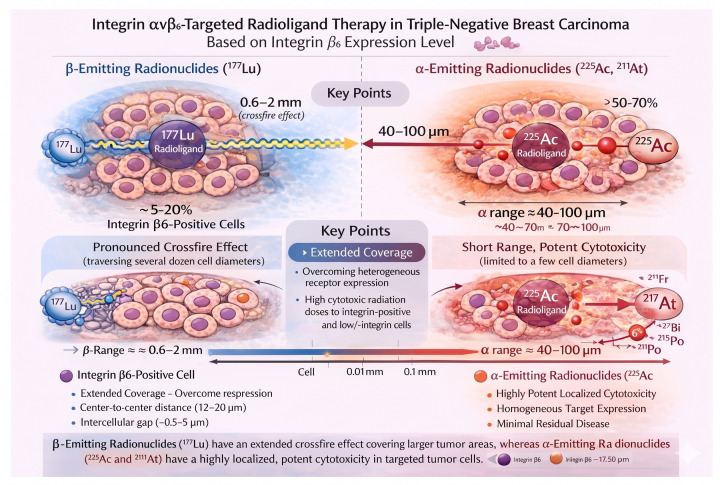
The figure illustrates comparisons of β-particle (^177^Lu) and α-particle (^225^Ac, ^211^At) radionuclides in relation to radioligand therapy directed against the integrin αvβ_6_ in the case of triple-negative breast carcinoma. The β-particles (^177^Lu) offer range variability within 0.6 to 2 mm, which provides multiple-dose delivery through a pronounced crossfire effect due to heterogeneous expression of the integrin αvβ6, and therefore, the ability to promote lethal effects to positive and negative/low-integrin-expressing cells. In the case of the α-emitters (^225^Ac, ^211^At), these demonstrate ≈40 to 100 μm (very few cell diameters) short range, and therefore, while being very powerful, these emitters possess limited cytotoxicity that is effective only for cells that are homogeneously targetable with a minimal number of cells remaining. Scale markers are: single cell ≈ 0.01 mm; distance of 0.1 mm, 10.0 μm. This figure was generated with the assistance of GPT-5.5 version, all scientific content depicted in Figure 6 accurately reflects the authors’ original work and interpretations. The use of AI was limited to visualization support and did not influence the scientific conclusions.

A key determinant of therapeutic efficacy in radionuclide therapy is the spatial distribution of radiation dose within the tumor microenvironment, which is governed by the relationship between tumor cell dimensions and radionuclide particle range [34]. Breast cancer epithelial cells typically measure approximately 10–20 µm in diameter, with center-to-center distances of ~12–20 µm and intercellular gaps of ~0.5–5 µm depending on tumor architecture [35,36]. Within this structural context, β-particles emitted by ^177^Lu, with a mean penetration range of approximately 0.6–0.7 mm in soft tissue (maximum ≈ 2 mm), are capable of traversing distances corresponding to several dozen tumor cell diameters. This physical property generates a pronounced cross-fire effect, enabling irradiation of tumor cells that do not directly bind the radioligand and thereby partially overcoming intratumoral heterogeneity of target expression (Figure 5). In contrast, α-particles emitted during the decay of ^225^Ac have a markedly shorter range of approximately 40–90 µm-equivalent to only a few cell diameters, but deposit very high LET, resulting in highly localized and potent cytotoxicity through the induction of irreparable DNA double-strand breaks (Figure 6).

Integrating these radiophysical considerations with radiobiological estimation of tumor architecture suggests that β-emitting radionuclides may achieve effective tumor-wide cytotoxicity even when integrin- positive cells constitute only a limited fraction (~5–20%) of the tumor cell population, owing to the extensive cross-fire effect. In contrast, α-emitting radionuclides likely require substantially higher target expression levels (≥50–70%) to achieve comparable tumor coverage, but may provide superior efficacy in settings of homogeneous target expression or minimal residual disease. Notably, our finding that 44% of TNBC cases exhibit moderate to strong integrin αvβ6 expression suggests that a clinically meaningful subset of tumors may be amenable to integrin-targeted radioligand therapy. In particular, β-emitter-based approaches may be effective even in tumors with heterogeneous expression patterns, whereas α-emitter-based strategies may be optimally suited for tumors with high and more uniform target expression or for the eradication of micrometastatic disease.

From a mechanistic perspective, these complementary radiobiologic properties support a model in which integrin-positive tumor cells act as focal sources of radiation following radioligand binding. The emitted particles—particularly from β-emitting radionuclides—can irradiate adjacent tumor cells with low or absent integrin expression, thereby amplifying the therapeutic effect beyond the directly targeted cell population. Conversely, the highly localized energy deposition of α-particles enables efficient cell killing at the single-cell level, which may be particularly advantageous for eliminating isolated tumor cells or treatment-resistant clones. Together, these features provide a strong radiobiologic rationale for the development of αvβ6-directed radiotheranostic strategies in TNBC and highlight the importance of matching radionuclide properties to tumor biology.

**Figure 7:**
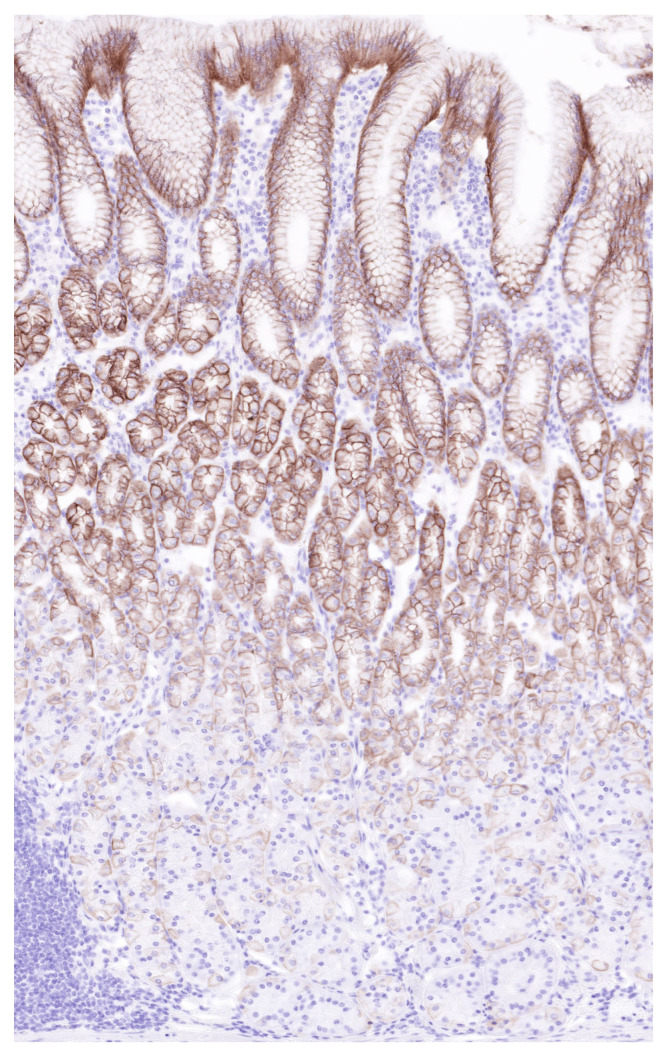
Immunostaining of the normal gastric mucosa. Gastric mucosa exhibits membranous integrin expression, with intensity increasing toward the upper layers of the mucosa (100×).

Importantly, the clinical translation of integrin αvβ6-targeted radionuclide therapy will require careful consideration of potential on-target, off-tumor effects. Although αvβ6 expression is relatively restricted in normal adult tissues, it has been reported in specific epithelial compartments, including the gastric mucosa (Figure 7) and small intestines [37]. These sites may therefore be at risk for unintended radiation exposure, particularly in the context of high-affinity radioligands. Comprehensive evaluation of biodistribution, tracer uptake, and organ-specific dosimetry will be essential to define the therapeutic window and identify potential dose-limiting organs. In this regard, the integration of immunohistochemistry with molecular imaging and dosimetric modeling may provide a robust framework for optimizing patient selection and treatment planning.

Collectively, our findings establish integrin αvβ6 as a promising diagnostic biomarker and therapeutic target in TNBC and provide a strong biological and radiobiological rationale for the further development of αvβ6-targeted radiotheranostic strategies. Beyond TNBC, these observations may also have broader relevance for other epithelial malignancies characterized by integrin αvβ6 expression. Future studies integrating histopathologic assessment, molecular imaging, and clinical outcomes will be essential to validate integrin αvβ6 as a predictive biomarker and to define its role in guiding precision radionuclide therapy.

Nevertheless, potential on-target off-tumor effects must be considered when targeting integrin αvβ6, as a physiological expression of this receptor has been reported in certain normal epithelial tissues. Histopathologic studies have demonstrated that integrin αvβ6 can be detected at low levels in epithelial compartments of the gastrointestinal tract, including the gastric mucosa, particularly in the context of epithelial turnover or tissue remodeling. In contrast, most quiescent adult tissues show minimal or absent αvβ6 expression, suggesting a potentially favorable therapeutic window for αvβ6-targeted imaging and radiotheranostic approaches. However, physiologic tracer uptake has frequently been observed in structures such as the pituitary gland and choroid plexus on integrin PET imaging, despite limited evidence for biologically relevant integrin αvβ6 expression in these tissues [38]. This apparent signal is most likely explained by nonspecific physiologic mechanisms rather than receptor-mediated binding. Both the pituitary gland and the choroid plexus are highly vascularized structures characterized by fenestrated capillaries and increased vascular permeability, which can facilitate tracer delivery and transient retention independent of receptor expression. In addition, the choroid plexus is located within cerebrospinal fluid-filled ventricular spaces, where radiotracer persistence and spill-in effects may contribute to apparent PET activity. Similar physiologic uptake patterns have been reported for several other PET tracers and therefore do not necessarily indicate true target expression. Consequently, although the restricted distribution of integrin αvβ6 in normal tissues supports the feasibility of αvβ6-targeted radiotheranostics, careful evaluation of physiologic tracer uptake, biodistribution, and radiation dosimetry will be essential to distinguish nonspecific accumulation from receptor-mediated uptake and to identify potential dose-limiting organs in future clinical applications [39,40,41].

In addition to its therapeutic implications, the identification of integrin αvβ6 expression by immunohistochemistry may have direct relevance for molecular imaging and disease staging. In this context, αvβ6-targeted PET using radiotracers such as ^68^Ga-Trivehexin represents a promising noninvasive approach for the detection and characterization of integrin- expressing tumors. Given the concordance between tissue-based integrin αvβ6 expression and target availability for ligand binding, tumors demonstrating moderate to strong expression by immunohistochemistry are likely to exhibit sufficient tracer uptake to enable sensitive in vivo imaging. Such imaging strategies may facilitate whole-body assessment of disease burden, improve staging accuracy, and allow for the identification of previously unrecognized metastatic lesions. Moreover, the integration of immunohistochemical profiling with αvβ6-targeted PET imaging may provide a robust framework for patient selection and treatment monitoring in radiotheranostic applications, thereby further advancing precision oncology approaches in TNBC and other integrin-positive tumors [37].

Collectively, our findings provide a strong biological and radiobiological rationale for the further development of αvβ6-targeted radiotheranostic strategies in triple-negative breast carcinoma. Beyond this disease context, these results may also have broader relevance for other epithelial malignancies and support the evaluation of integrin αvβ6 as a predictive biomarker for patient selection in precision radionuclide therapy.

There are some limitations of this study that need to be addressed. First, the sample size is relatively small (*n* = 48) and from a single institution, which may limit the generalizability of the findings and preclude subgroup analyses based on clinicopathological features. Second, the retrospective design is subject to potential selection bias and the use of core needle biopsy specimens only. Third, although the semi-quantitative immunohistochemical scoring was performed by experienced pathologists blinded to clinical data, the subjective nature of the scoring is inherently associated with interobserver variability, which may affect reproducibility between different evaluators. Finally, the absence of an independent validation cohort and lack of correlation with clinical outcome (e.g., progression-free survival, response to therapy) mean that the predictive value of integrin αvβ6.

## 5. Conclusions

Although the present findings are exploratory, they establish a foundation for future prospective studies integrating immunohistochemical profiling, in vivo imaging, and evaluation of therapeutic response. Further investigation is warranted to determine whether integrin αvβ6 expression predicts radioligand uptake, treatment efficacy, and clinical outcomes across both primary and metastatic disease. Collectively, these findings highlight integrin αvβ6 as a promising biomarker and potential therapeutic target in triple-negative breast carcinoma and potentially other carcinoma entities characterized by unmet therapeutic needs.

## Figures and Tables

**Table 1:** Integrin αvβ6 expression levels were assessed by immunohistochemistry on core biopsies from 48 TNBC patients.

Integrin α_v_β_6_ Expression Score Category	Cases (*n* = 48)	Percentage
Negative	16	33.3%
Weak	11	22.9%
Moderate	9	18.8%
Strong	12	25%

## Data Availability

The data presented in this study are available from the corresponding author upon reasonable request.
